# Investigation of the Association between Sleep Disorders with Subsequent Depression in Children and Adolescents—A Retrospective Cohort Study with 62,796 Patients

**DOI:** 10.3390/children11070758

**Published:** 2024-06-22

**Authors:** Nimran Kaur, Céline Vetter, Marcel Konrad, Karel Kostev

**Affiliations:** 1 Epidemiology, IQVIA, Bangalore 560 103, India; 2 Epidemiology, IQVIA, 60549 Frankfurt am Main, Germany; 3 Health & Social, FOM University of Applied Sciences for Economics and Management, 60486 Frankfurt am Main, Germany; 4 University Clinic, Philipps-University, 35043 Marburg, Germany

**Keywords:** sleep disorders, depression, children, retrospective cohort, Germany

## Abstract

Background: Poor quality of sleep is a widespread issue in modern society, and even children are being diagnosed with sleep disorders nowadays. Sleep disruption in children can lead to poor mental health in the long term. The present study aimed to evaluate the association between sleep disorders and subsequent depression in children and adolescents. Methods: This retrospective cohort study used electronic medical records from the IQVIA^TM^ Disease Analyzer database. It included children and adolescents aged 6–16 with an initial diagnosis of a sleep disorder and age- and gender-matched patients without sleep disorders treated by one of 274 office-based pediatricians in Germany between January 2010 and December 2022. The five-year cumulative incidence of depression in the cohorts with and without sleep disorders was studied with Kaplan–Meier curves using the log-rank test. Multivariable Cox regression analyses were used to assess the association between sleep disorders and depression. Results: The present study included 10,466 children and adolescents with and 52,330 without sleep disorder diagnosis (mean age 10 ± 3 years, 48% female). Within five years after the index date, 5% of sleep disorder patients and 2% of the matched non-sleep disorder cohort had been diagnosed with depression. A strong and significant association was observed between sleep disorders and subsequent depression (HR: 2.34; 95% CI: 2.09–2.63). This association was stronger in adolescents (HR: 3.78; 95% CI: 3.13–4.56) than in children. Upon the exclusion of depression diagnoses in the first year after the index date, the association between sleep disorders and depression remained strong and significant (HR: 1.92; 95% CI: 1.68–2.19). Conclusions: This study indicates a strong and significant association between sleep disorders and depression.

## 1. Introduction

Quality sleep is required for sound physical and mental health. Furthermore, children live in a multi-dimensional ecosystem and might be unable to control their sleep. Extensive scientific data suggest that assessment for sleep health should be on par with examination and treatment for other health conditions by healthcare professionals [1]. A study conducted in 2022 [2] observed that the proportion of two- to six-year-olds who suffered from sleep disorders had nearly doubled by 2021 (7.8%) compared to 2001 [3]. More and more children are being diagnosed with sleep disorders (problems falling or staying asleep, obstructive sleep apnea, sleepwalking, other parasomnias, and daytime symptoms), which is worrisome. Furthermore, twelve percent of European school-attending children demonstrated at least one sleep-related problem every night: concentration difficulties, daytime fatigue, daytime naps, nightmares, night terrors, sleepwalking, and nocturnal awakening [4,5]. About 5% of the children struggled with falling asleep, staying asleep, nocturnal awakening, and parasomnias [6].

Schlarb et al. [7] surveyed German children and highlighted sex- and age-related differences in the progression and the burden of sleep problems. Sleep problems were reported among 22.6% of zero- to two-year-olds and about 16% of children between three and six years of age. Parents of three- to six-year-olds reported that their children had trouble falling asleep (9%), problems with staying asleep through the night (9.4%), or a combination of both of these difficulties (2.7%). School-attending children (aged 7 to 10 years) were reported to present with increased problems in falling asleep (13.6%) and staying asleep through the night (4.9%). Adolescents (11 to 13 years) showed the highest prevalence of sleep problems (23.1%) among all age groups, reporting difficulties falling asleep (17%) and problems sleeping through the night (7.8%). Older (14- to 17-year-old) children showed stark sex-related differences, i.e., girls had a higher prevalence of sleep problems and a statistically significant risk of developing sleep problems compared to boys. Children (14 to 17 years) suffered from difficulties falling asleep (higher among girls (17.6%) than boys (15.1%)) and problems in sleeping through the night (higher among girls (11%) than boys (5.1%)). Sleep disorders among children can cause long-term effects like attention issues, impaired learning capacity, impaired cognitive functioning, and poor performance in school [8]. 

There is convincing research on the relationship between somatic health and mental health issues, such as anxiety, depression, somatization, physical distress, etc., among 15- to 95-year-olds [9,10]. Seven in ten children suffer from global sleep disturbances, and the correlations between sleep problems and emotional or behavioral problems are statistically significant [2,11,12]. A meta-analysis of 16 prospective cohort studies conducted including 5- to 24-year-olds found a statistically significant association between sleep disruption and depressive symptoms in children and youths [13]. A prospective study that followed children aged 1.5 years until they turned eight years old established a reciprocal relationship between sleep and internalizing or depressive symptoms [14]. A narrative review concluded that children with depression could exhibit an array of sleep problems, which are associated with severe depression, exaggerated levels of fatigue, suicidal ideation, decreased concentration, undefined pain, and physical ailments [15]. 

One problem with today’s lifestyle is that many children are not physically active. For example, previous research demonstrated that physical activity had a positive effect on the sleep quality of children [16,17]. Lack of physical activity, on the other hand, could lead to poor sleep quality and psychological problems. 

Although the results of published studies on the association between sleep quality and physical problems are critical, they are subject to several limitations. Most of the studies were conducted outside of Europe, included small samples, or used different methods and settings. The aim of the current study was, therefore, to evaluate the association between sleep disorders and subsequent depression in children and adolescents (6–16 years). As mentioned in a nationally representative survey, mental health disorders are vastly underestimated, undiagnosed, and untreated in Germany [5]; hence, generating data that emphasize the need to focus on this area is of the utmost importance. 

## 2. Methods

### 2.1. Database

This retrospective cohort study used data from the IQVIA^TM^ Disease Analyzer database, which contains anonymous electronic medical records, including baseline demographic data, diagnoses, and prescriptions from computer systems used in office-based practices [18]. The panel of practices included in this database is representative of general and specialized practices in Germany [18]. This data source has often been used in previous studies focusing on children’s health [19].

### 2.2. Study Population

**Figure 1:**
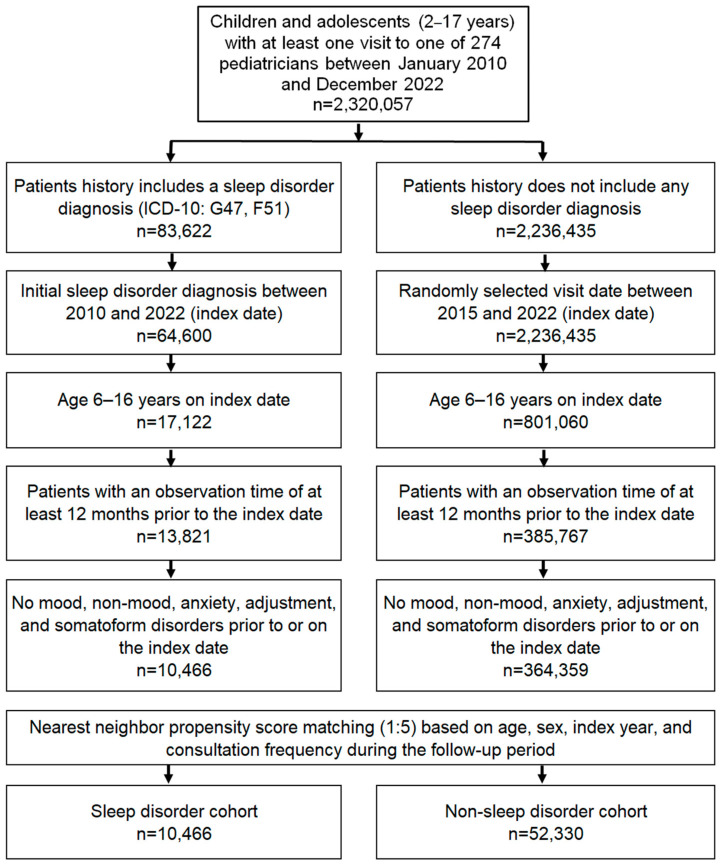
Selection of study patients.

This study included children and adolescents aged 6–16 with an initial diagnosis of sleep disorder (ICD-10: G47, F51) from 274 office-based pediatricians in Germany between January 2010 and December 2022 (index date; Figure 1). The age limit was 16 for at least one year of follow-up (pediatricians in Germany do not treat patients from the age of 17 and above). Patients were only included when they had an observation time of at least 12 months before the index date and had not been diagnosed with any mood (ICD-10: F30–F39), non-mood (ICD-10: F20–F29), anxiety (ICD-10: F41), adjustment (ICD-10: F43), or somatoform (ICD-10: F45) disorders before or on the index date. After applying similar inclusion criteria, children and adolescents without sleep disorder diagnoses were matched to those with sleep disorder diagnoses using nearest neighbor propensity score matching (1:5) based on age, sex, index year, and average yearly consultation frequency during the follow-up. For the non-sleep disorder cohort, the index date of a randomly selected visit was between January 2010 and December 2022 (Figure 1). 

### 2.3. Study Outcomes 

The primary outcome of this study was the initial diagnoses of depression (ICD-10: F32, F33) in the up to five years following the sleep disorder index date. As this study was a sensitivity analysis, only depression diagnoses up to 1 year before and up to 5 years after the index date were analyzed. This sensitivity analysis tested the robustness of our findings, as it is well established that sleep disorders may also be a symptom of depression [20]. The last day in the database was 31 December 2023. 

### 2.4. Statistical Analyses

Differences in the sample characteristics and diagnosis prevalence between cohorts with versus without sleep disorders were compared using the Wilcoxon signed-rank test for continuous variables and the McNemar test for categorical variables. The five-year cumulative incidence of depression in the cohorts with and without sleep disorders was examined with Kaplan–Meier curves using the log-rank test. Multivariable Cox regression analyses were conducted to assess the association between sleep disorders and depression, adjusted for most common co-diagnoses documented within 12 months prior to the index date, including pain–related diseases (back pain: ICD-10: M54, fibromyalgia: ICD-10: M79, rheumatoid arthritis: ICD-10: M05, M06, headache: ICD-10: G43, G44, R51, undefined pain: ICD-10: R52), obesity (ICD-10: E66), vasomotor and allergic rhinitis (ICD-10: J30), chronic rhinitis, nasopharyngitis, and pharyngitis (ICD-10: J31), chronic sinusitis (ICD-10: J32), asthma (ICD-10: J45), dermatitis (ICD-10: L30), developmental disorders of speech and language (ICD-10: F80), developmental disorder of motor function (ICD-10: F82), attention-deficit hyperactivity disorders (ICD-10: F90), and conduct disorders (ICD-10: F91). Cox regression analyses were conducted separately for two age groups (6–12 and 13–16 years), girls and boys. Due to the multiple comparisons involved, a *p*-value of <0.01 was considered statistically significant. Analyses were performed using SAS version 9.4 (SAS Institute, Cary, NC, USA).

## 3. Results

### 3.1. Basic Characteristics of the Study Sample

The present study included 10,466 children and adolescents with and 52,330 without sleep disorder diagnoses. The basic characteristics of the study patients are displayed in Table 1. The mean age was 10.1 (standard deviation (SD): 3.0 years), 76.2% were 6–12 years old, and ~48% were female. Patients visited their pediatricians an average of 4.3 times per year during the follow-up period. Patients with sleep disorders had a slightly higher prevalence of chronic rhinitis, nasopharyngitis, and pharyngitis, sinusitis, dermatitis, developmental disorders, attention-deficit hyperactivity, and conduct disorders (Table 1). The most frequent sleep disorder diagnosis was non-specified sleep disorder (ICD-10: G47.9, 54.9%), followed by insomnia (29.1%). The proportions for all other ICD-10 codes were small, between 1% and 6%.

### 3.2. Association between Sleep Disorders and Subsequent Depression

**Figure 2:**
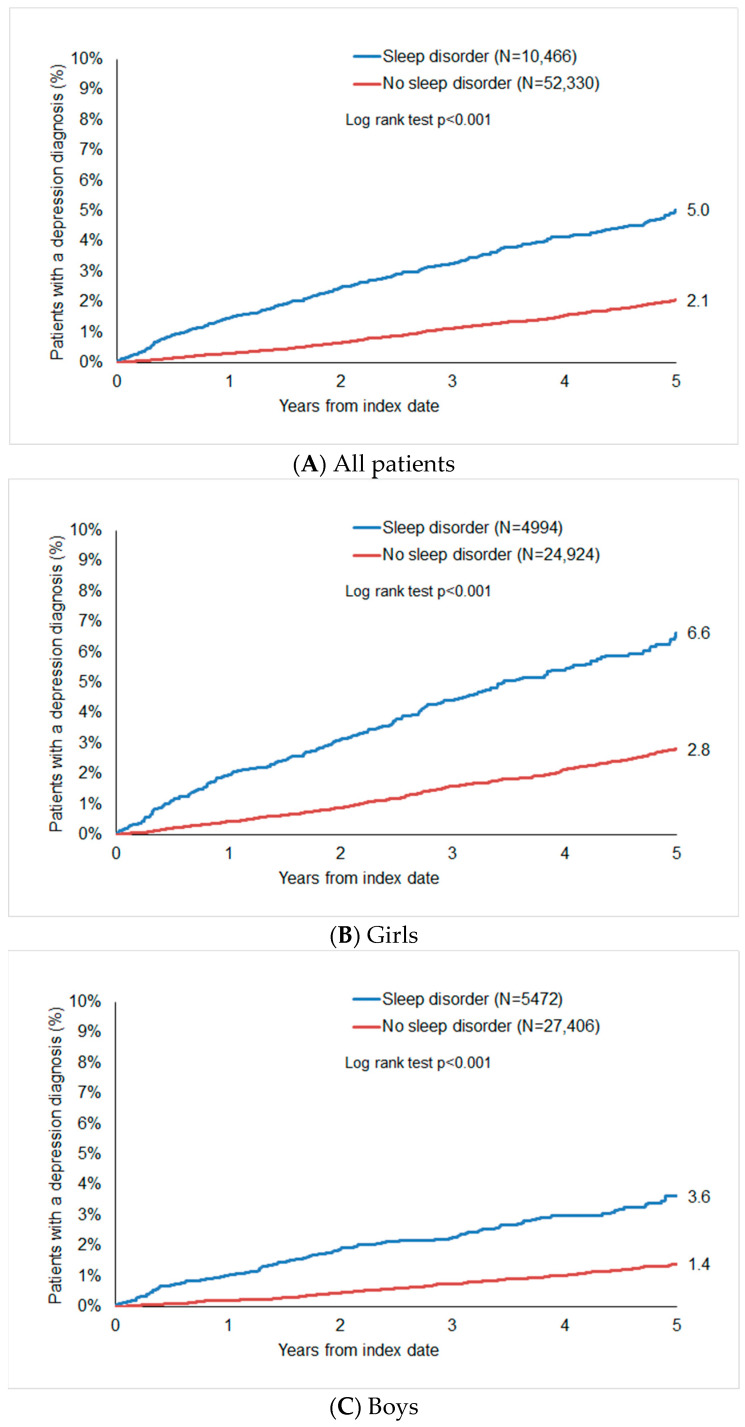
Cumulative incidence of depression in children and adolescents with and without sleep disorders.

Within five years after the index date, 5.0% of sleep disorder patients and 2.1% of the matched non-sleep disorder cohort (*p* < 0.001) had been diagnosed with depression (Figure 2A). The cumulative incidence of depression was higher in girls (6.6% vs. 2.8%, Figure 2B) than in boys (3.6% vs. 1.4%, Figure 2C).

The regression analysis showed a strong, significant association between sleep disorders and subsequent depression (HR: 2.34; 95% CI: 2.09–2.63) (Table 2), which was similar in girls (HR: 2.34; 95% CI: 2.03–2.70) and boys (HR: 2.32; 95% CI: 1.91–2.82). This association was stronger in adolescents (HR: 3.78; 95% CI: 3.13–4.56) than in children (HR: 1.80; 95% CI: 1.55–2.09). In sensitivity analyses excluding depression diagnoses in the first year after the index date, the association between sleep disorders and depression still was very strong and significant (HR: 1.92; 95% CI: 1.68–2.19) (Table 2).

## 4. Discussion

In this study, we report a substantial association of sleep disorders with subsequent depression, with a more significant effect size in adolescents than in children. The cumulative incidence of depression was higher in girls than in boys. 

Our findings align with existing evidence, showing that sleep disorders are associated with depression among children and adolescents [15]. A systematic review and meta-analysis (n = 74 studies) conducted among 361,505 adolescents showed reductions in positive states of moods following the loss of sleep, leading to intensification of depressive symptoms [21]. Secondly, the gradual delay in sleep timing caused by the delayed circadian rhythm throughout adolescent development further exacerbates the delay in the onset of sleep, increasing depression symptoms [22]. These misalignments of the biological clock should be noted in clinical practice to help diagnose depressive symptoms in this age group. A survey of adolescents aged 14 to 20 years reported that physiologically determined delayed sleep onset is conducive to psychological depression. The study also found that a more extended period between the adolescent’s bedtime and onset of sleep increases the chances of suffering from negative pre-sleep cognitions. This, in turn, leads to repetitive negative thinking, which is related to increased levels of depression among adolescents [23]. While other pathways explain the linkage between disturbed sleep and depression, for example, inflammatory pathways activation, transformed neuroplasticity, altered learning, or a disruption of the circadian rhythm [20], no definitive answers have been found. Future researchers may focus on specific sleep disorders and emotional behavioral problems to draw conclusive results. Prevention of depression should be targeted through multi-component risk profiles, and data synthesis is needed to identify the modifiable risk factors required to focus on and prioritize specific populations as part of a prevention plan. 

A systematic review of infants and children (0–12 years) observed physiological changes in sleep patterns from birth to early adulthood due to varied maturational stages of the brain and adjustment to the social environment [24]. Sleep duration reduces from nearly 14 h at six months to just 8 h at the age of 16 years [25]. The 2 h delay in sleep onset is understood as a typical part of puberty, which might be related to gonadal hormone changes [26]. The delay in sleep onset time, referred to as evening chronotype, manifests in younger girls rather than boys, consistent with sex-related differences in the onset of puberty. In addition, research claims that this ‘epidemic’ of sleep shortage might be caused by adolescents’ evening chronotype and early morning wake-up times, which differ from those of younger children [27]. The literature also suggests that decreased sleep in children may be due to increased screen media use during late hours as per their parents and existing studies [2,12,28,29]. An increased proportion of healthy children are exposed to insufficient sleep and altered sleep patterns. Similarly, our study findings emphasized that older children were more prone to depression with sleep deprivation than younger ones. Nevertheless, a systematic review and meta-analysis suggested that depression did not differ with respect to a child’s age at the baseline and concluded that children with sleep disorders are potentially vulnerable to depression later in life [13]. These differences might have been due to the pooled effect of the results in the former study. Numerous studies have highlighted a bidirectional association of sleep with internalizing symptoms (such as depression, anxiety, self-harm, suicidal intention, and other internalizing symptoms) [12,30]. This study emphasizes the value of a multi-pronged approach to understanding these phenomena. A meta-analysis involving 198,893 participants aged 12.5 ± 3.1 years to 48.1 ± 15.6 years found that sleep problems (nightmares and insomnia) increase suicidal ideation or attempts among depressed patients [31]. Wang et al. highlighted the need for interventions for depressed patients due to the grave potential consequences. Though disturbances in sleep can be effectively treated, scant randomized controlled trials have targeted the improvement of sleep for preventing depression among young children (<12 years) [32]. These circumstances suggest that more RCTs are needed, especially involving younger age groups, to prevent young people from becoming vulnerable to depression. 

The current study has several important strengths, including the documentation of both sleep and depression by pediatricians across Germany. The large representative sample of German children is a further strength. The current research includes several time-varying covariates, measured at all the same time points as the primary exposure. However, there are also several limitations worth noting. First, the current study did not include an in-depth assessment of other sleep disorders or aspects related to sleep hygiene, as well as information on methods used for diagnosis of sleep disorders. Future researchers may explore the association between sleep and a more comprehensive range of sleep-related disorders and behaviors. This sample was taken from an outpatient setting, so the findings may not be generalizable to hospital populations. Similarly, the present study does not address the developmental changes concerning sleep in children during this phase. Future research should observe these trajectories concerning sleep across the developmental period and aim to understand whether these associated behaviors change at different ages or between sexes. The database does not contain information on lifestyle-related factors (e.g., physical mobility), socio-economic factors, or other relevant variables. As with all large-scale longitudinal studies, missing data and attrition might impact this study over time. Misclassification bias might be present as both exposure and outcomes were either parent- or self-reported. 

## 5. Conclusions

This study concludes that there is a strong and significant association between sleep disorders and depression among children. Older boys and girls showed a greater prevalence of clinical depression. Our findings encourage the development of standardized and validated clinical tools to measure sleep disturbances among children that explicitly address several aspects of sleep quality. Sleep problems respond effectively to cost-effective interventions and are often readily identified and reported by parents in younger age groups [13]. Randomized control clinical trials that target children with disturbed sleep are required to plan and evaluate the efficacy of feasible depression prevention programs. The present study’s findings must be considered in the context of its limitations.

## Figures and Tables

**Table 1:** Baseline characteristics of the study sample (after propensity score matching).

Variable	Sleep Disorders Cohort (N = 10,466)	Matched Controls (N = 52,330)	*p*-Value
Age (Mean, SD)	10.1 (3.0)	10.1 (3.0)	0.958
Age 6–12	7972 (76.2)	39,881 (76.2)	0.930
Age 13–17	2494 (23.8)	12,449 (23.8)	
Female	4994 (47.7)	24,924 (47.6)	0.869
Male	5472 (52.3)	27,406 (52.4)	
Number of physician visits per year during the follow-up (Mean, SD)	4.3 (3.3)	4.3 (3.3)	1.000
Index year			
2010–2013	2180 (20.8)	11,872 (22.7)	
2014–2016	2554 (24.4)	12,506 (23.9)	0.004
2017–2019	2770 (26.5)	13,671 (26.1)	
2020–2022	2962 (28.3)	14,281 (27.3)	
Diagnoses documented within 12 months prior to or on index date			
Pain-related disorders	1744 (16.7)	6066 (11.6)	<0.001
Obesity	1003 (9.6)	4734 (9.1)	0.082
Vasomotor and allergic rhinitis	1512 (14.5)	7968 (15.2)	0.042
Chronic rhinitis, nasopharyngitis, and pharyngitis	2588 (24.7)	12,127 (23.2)	<0.001
Chronic sinusitis	791 (7.6)	2776 (5.3)	<0.001
Asthma	1259 (12.0)	7144 (13.7)	<0.001
Dermatitis	2848 (27.2)	12,756 (24.4)	<0.001
Developmental disorders of speech and language	3687 (35.2)	15,985 (30.6)	<0.001
Developmental disorders of motor function	1292 (12.3)	5229 (10.0)	<0.001
Attention-deficit hyperactivity disorders	1385 (13.2)	3953 (7.6)	<0.001
Conduct disorders	954 (9.1)	2109 (4.0)	<0.001

Proportions of patients given in N and % unless otherwise indicated. SD: standard deviation.

**Table 2:** Association between sleep disorders and subsequent depression in children and adolescents followed by office-based pediatricians in Germany (multivariable Cox regression models).

	Depression Diagnosis within Five Years after the Index Date *p*-Value		Depression Diagnosis in the Time > 1–5 Years after the Index Date *p*-Value	
Patient Group	HR (95% CI)	*p*-Value	HR (95% CI)	*p*-Value
Total	2.30 (2.06–2.58)	<0.001	1.89 (1.66–2.16)	<0.001
Age 5–12	1.78 (1.54–2.07)	<0.001	1.68 (1.44–1.97)	<0.001
Age 13–16	3.73 (3.08–4.50)	<0.001	2.74 (2.12–3.55)	<0.001
Girls	2.29 (1.99–2.65)	<0.001	1.92 (1.62–2.26)	<0.001
Boys	2.30 (1.89–2.79)	<0.001	1.86 (1.48–2.32)	<0.001

## Data Availability

The datasets used and analyzed during the current study are available from the corresponding author upon reasonable request. The data are not publicly available due to privacy reasons.
